# Isolation and characterization of bacteriophages specific to *Streptococcus equi* subspecies *zooepidemicus* and evaluation of efficacy *ex vivo*

**DOI:** 10.3389/fmicb.2024.1448958

**Published:** 2024-10-28

**Authors:** Martin Köhne, Ronja Hüsch, Anna Tönissen, Matthias Schmidt, Mathias Müsken, Denny Böttcher, Juliane Hirnet, Madeleine Plötz, Sophie Kittler, Harald Sieme

**Affiliations:** ^1^Unit for Reproductive Medicine – Clinic for Horses, University of Veterinary Medicine, Foundation, Hannover, Germany; ^2^Department of Technical Biogeochemistry, Helmholtz Centre for Environmental Research –UFZ, Leipzig, Germany; ^3^Central Facility for Microscopy, Helmholtz Centre for Infection Research – HZI, Braunschweig, Germany; ^4^Institute for Veterinary Pathology, Faculty of Veterinary Medicine, Leipzig University, Leipzig, Germany; ^5^Institute of Food Quality and Food Safety, University of Veterinary Medicine, Foundation, Hannover, Germany

**Keywords:** bacteriophage, uterus, *Streptococcus equi* subspecies *zooepidemicus*, *ex vivo* model, non-antibiotic treatment

## Abstract

*Streptococcus (S.) equi* subspecies (subsp.) *zooepidemicus* is an important facultative pathogen in horses and can cause severe infections in other species including humans. Facing the post-antibiotic era, novel antimicrobials are needed for fighting bacterial infections. Bacteriophages (phages) are the natural predators of bacteria and discussed as a promising antimicrobial treatment option. The objective of this study was to isolate and characterize *S. equi* subsp. *zooepidemicus-*specific phages for the first time and to evaluate their efficacy *in vitro* and *ex vivo*. In total, 13 phages with lytic activity were isolated and host ranges were determined. Two phages with broad host ranges and high efficiency of plating (vB_SeqZP_LmqsRe26-2 (lytic activity: 30/37 bacterial isolates) and vB_SeqZP_LmqsRe26-3 (lytic activity: 29/37 bacterial isolates)) and one phage with relatively low efficiency of plating (vB_SeqZP_LmqsRe26-1) were selected for further characterization, including electron microscopy and whole genome sequencing. In *in vitro* planktonic killing assays at two tested multiplicities of infection (MOI 1 and MOI 10), significant bacterial growth reduction was observed when the phages vB_SeqZP_LmqsRe26-2 and vB_SeqZP_LmqsRe26-3 were added. These phages were subsequently co-incubated with clinical *S. equi* subsp. *zooepidemicus* isolates in an equine endometrial explant model but did not achieve bacterial growth reduction at MOI 1 and MOI 10. However, helium ion microscopy revealed presence of particles adherent to the bacteria on the explant after incubation (25 h), suggesting possible phage-bacteria interactions. In conclusion, phages against *S. equi* subsp. *zooepidemicus* were successfully isolated and characterized. Promising results were observed in *in vitro* but no significant reduction was detected in *ex vivo* experiments, requiring additional investigations. However, after further adaptations (e.g., optimization of MOIs and phage administration or use of phage-antibiotic combination), phages could be a potential antimicrobial tool for future therapeutic use in *S. equi* subsp. *zooepidemicus* infections, although the available results do not currently support the therapeutic usage.

## 1 Introduction

*Streptococcus (S.) equi* subspecies (subsp.) *zooepidemicus* is a major causative agent of infections in foals and adult horses (Timoney, 2004), leading to severe respiratory diseases (Oikawa et al., 1994) and infection of inner organs, such as liver, lung, brain, kidney as well as joints (Timoney, 2004). In foals, *S. equi* subsp. *zooepidemicus* infection is a frequent cause for neonatal bacterial sepsis (Sanchez et al., 2008). In equine reproductive medicine, *S. equi* subsp. *zooepidemicus* is the bacterial species most often detected in bacterial endometritis cases (Köhne et al., 2024b; Rathbone et al., 2023; Ricketts, 2011). However, *S. equi* subsp. *zooepidemicus* is also detected in cases of necrotizing myositis (Kittang et al., 2017), meningitis (Eyre et al., 2010) and arthritis (Friederichs et al., 2010) in humans after direct horse-human contact (Pelkonen et al., 2013) or consumption of contaminated food (Bordes-Benítez et al., 2006). Furthermore, it plays a role as opportunistic pathogen in a large variety of domestic animals including cats (Blum et al., 2010), dogs (Priestnall and Erles, 2011), mice (Literák and Mráz, 1991), pigs (Feng and Hu, 1977) and small ruminants (Steward et al., 2017).

In equine medicine, the condition of bacterial endometritis has been rated as one of the major clinical problems (Traub-Dargatz et al., 1991) and is a leading cause for reproductive failure (Hurtgen, 2006; Riddle et al., 2007; LeBlanc and Causey, 2009). Traditionally, the treatment of bacterial endometritis involves antibiotic drugs, which are administered either locally or systemically (Canisso et al., 2020; Köhne et al., 2020). In addition alternative therapeutic approaches with antibacterial properties, e.g., ozone (Köhne et al., 2023; Ávila et al., 2022) and hydrogen peroxide (Ferris et al., 2016), have been investigated *in vitro* and *in vivo* with varying degrees of success. Searching for novel antimicrobial treatment options against bacterial infections is urgently needed, considering the increasing numbers of multidrug-resistant (MDR) bacteria worldwide (Murray et al., 2022) and the presence of penicillin-resistant *S. equi* subsp. *zooepidemicus* in cases of equine bacterial endometritis (Pisello et al., 2019; Davis et al., 2013).

A promising antimicrobial approach for treating bacterial infections is the use of bacteriophages (phages) that has received increasing attention in medical research as well as in the general public (Kortright et al., 2019). Phages are viruses that exclusively infect bacterial cells, having very specific bactericidal activity against their host bacteria and leaving the physiological microbiome unaffected (Kasman and Porter, 2022; Kutateladze and Adamia, 2010). Recently, regulatory hurdles for using phages in veterinary antibacterial therapy have been reduced in the European Union (European Commission, 2019; European Medicines Agency, 2023) and phage products could be approved for veterinary medicine in the near future (Peh et al., 2023). Studies from human (Van Nieuwenhuyse et al., 2022) as well as veterinary medicine (Smith and Huggins, 1983) reported the successful use of phages in antibacterial therapy. However, promising results of *in vitro* experiments for large animal gynecology (Bicalho et al., 2010), using phages specific to *Escherichia coli* isolates associated with bovine metritis, have not been transferred into a clinical study (Machado et al., 2012). In equine medicine, studies on phages specific against equine pathogens, e.g., *Salmonella enterica* subsp. *enterica* serovar Abortusequi (Wang et al., 2020) and equine keratitis-associated *Pseudomonas aeruginosa* (Furusawa et al., 2016), have been carried out in mouse models only and have not been published for species-specific models nor *in vivo* experiments to date.

In contrast to non-target-species animal models, *ex vivo* models are suitable for studying phage efficacy while reducing animal experimentation. These models provide an environment that mimics the situation in the target organ of the target species (Thompson et al., 2020b) without using live animals. For the equine uterus, several *ex vivo* models, including isolated-perfused uteri (Köhne et al., 2022; Köhne et al., 2024a), organoid (Thompson et al., 2020a) and explant cultures (Nash et al., 2008; Monteiro de Barros et al., 2021) have been introduced and validated. In this study, an explant culture model has been selected since it provides access to the endometrial surface – contrasting endometrial organoids – and allows for the replication of experiments using one uterus, which cannot be achieved in the isolated-perfused uterus model.

To our knowledge, no phages specific to *S. equi* subsp. *zooepidemicus* have been introduced in collections for research and therapeutic purposes despite the zoonotic potential of their host and the demands for reduction of antibiotic drugs in veterinary medicine.

Hence, the objectives of the present study were to (i) isolate and characterize *S. equi* subsp. *zooepidemicus*-specific phages for the first time and (ii) to determine their bactericidal capacities *in vitro* and in an explant model, including visualization of possible phage-bacteria interactions on the endometrium.

## 2 Materials and methods

### 2.1 Bacterial isolates

*Streptococcus equi* subsp. *zooepidemicus* isolates (*n* = 37) from horse-derived samples (Supplementary Table S1) were provided by the Institute of Microbiology, University of Veterinary Medicine Hannover, Foundation, Germany and Labor Dr. Böse, GmbH, Harsum, Germany. The isolates were stored in cryotubes (Carl Roth GmbH + Co. KG, Karlsruhe, Germany) at −80°C. Before starting the experiments, isolates were plated out on Columbia agar supplemented with sheep blood (Oxoid Deutschland GmbH, Wesel, Germany) and incubated aerobically at 37°C overnight.

The study has been approved by the institutional review board (Doctoral Commission, Stiftung Tierärztliche Hochschule Hannover, 2023, 3.5) and the animal welfare officer (TVO-2023-V-18) as ethical.

### 2.2 Phage isolation and propagation

The soft-agar overlay technique was used to isolate *Streptococcus* phages from environmental samples as described previously (Shakeri et al., 2021; Steffan et al., 2021). Phages were isolated from different horse associated samples (manure, uterine lavage fluid, drain water of horse husbandries; *n* = 12). Two grams of each sample were dispersed in sodium chloride-magnesium sulfate (SM) buffer (100 mM NaCl, 8 mM MgSO_4_, 50 mM Tris–HCl (pH 7.5)) in falcon tubes (50 mL). Next, the dispersed samples were centrifuged (5,000 × *g*, 20 min, 4°C) and the supernatant was subsequently filtrated through a 0.2 μm polyethylene sulfone membrane (PES) syringe filter (Carl Roth GmbH + Co. KG, Karlsruhe, Germany). Subsequently, the presence of phages in the filtrate was tested using the soft agar-overlay method. In brief, overnight cultures of *S. equi* subsp. *zooepidemicus* isolates (*n* = 6) were prepared on Columbia agar supplemented with sheep blood [Oxoid Deutschland GmbH, Wesel, Germany; incubation (37°C, 18 h)]. For each isolate, a McFarland standard 3.0 was prepared and 100 μL of the bacterial suspension was transferred to a liquefied LB agar overlay suspension (0.7% agar agar) at 48°C. Next, 100 μL of filtered dispersed samples (*n* = 12) was added and the overlay was poured onto LB agar plates (1.5% agar agar) in duplicates. After incubation at room temperature for 10 min, double-agar-overlays were incubated aerobically for 24 h at 37°C. Presence of lytic phages was assumed if lytic areas, so called plaques, were present. The phages were then isolated and purified by a successive 3-fold picking and plating procedure of single plaques. Afterwards, the phages were propagated to obtain concentrations of 10^8^–10^9^ PFU/ml and stored at 4°C until further use. The phage titers were determined by plating 100 μL of a 10-fold serial dilution series of the phage suspension on the respective host bacterial isolate using the soft agar overlay technique as described above.

### 2.3 Host range and efficiency of plating

The host range of the isolated phages (*n* = 13) was determined in accordance with the plaque assay method of Korf et al. (2020), with some modifications, while susceptibility of the bacteria was indicated by efficiency of plating (EOP), as calculated and presented by Steffan et al. (2021). In short, LB-agar plates were overlaid with 5 mL LB soft agar, containing 100 μL of the respective phage suspension (10^6^ – 10^9^ PFU/ml) and 100 μL of the *Streptococcus equi* subsp. *zooepidemicus* isolate (10^8^ CFU/mL; *n* = 37). After aerobic incubation at 37°C for 18 h, the sensitivity of the tested bacteria to the phages was determined by counting the number of plaques in the bacterial lawns. All combinations were tested in triplicate. The relative efficiency of plating was graded according to the presence of a zone of inhibition or number of plaques visible on the agar plates: no sensitivity (no plaque formation), moderately low sensitivity (presence of an opaque plaque), low sensitivity (EOP < 0.1), moderate sensitivity (0.1 ≤ EOP ≤ 1), high sensitivity (1 < EOP ≤10), ultimate sensitivity (10 < EOP). EOP of 1 represented similar plaque formation as observed in the original host bacterial isolate.

### 2.4 Extraction of phage DNA, whole-genome sequencing, and bioinformatics analysis

For further characterization, phages (*n* = 3) were selected according to their host range and EOP (broad host range: *n* = 2; low EOP: *n* = 1). Three hundred ml of phage suspensions (10^8^ – 10^10^ PFU/ml) were prepared as described above. Subsequent to filtration, the suspensions were centrifuged (24,000 × *g*, 2 h, Avanti J-26S XP, Beckmann Coulter Inc., Brea, United States) and the resulting pellets were resuspended with a small amount of SM-buffer and purified by CsCl-gradient (165,100 × *g*, 4°C, 2 h, Optima XPN-100, Beckmann Coulter Inc., Brea, United States). Thereafter, cesium chloride was removed from the phages via dialysis with SM-buffer. The resulting phage suspensions were used for both, electron micrographs and DNA isolation. Phage DNA was extracted from the purified virions treated with Norgen’s RNase-Free DNase I Kit (Norgen Biotek Corp., Canada) and Phage DNA Isolation Kit (Norgen Biotek Corp., Canada) was used for DNA extraction and according to the manufacturer’s instructions. The concentration of phage DNA molecules was determined using ds DNA HS Assay Kit (Life Technologies Corporation, Oregon, United States) before submission to whole genome sequencing using a Nextseq sequencing system (Microsynth AG, Balgach, Switzerland). For Phage LmqsRe26-2 long read sequencing was performed using a MinIon according to the manufactures instructions. Assembly and annotation was performed using the Galaxy platform (The Galaxy Community, 2022). Contigs were assembled using the SPAdes software (St. Petersburg genome assembler; version: 3.15.5) or Flye (Version 2.9.4). Phage termini were determined using PhageTerm (Garneau et al., 2017) and confirmed by PCR as described by Boeckman et al., 2024. Homology with previously published bacteriophages was detected by BLASTN (Basic Local Alignment Search Tool, http://www.ncbi.nlm.nih.gov Version 2.15.0; Lin et al., 2016). Annotation of CDS was conducted with Prokka (Prokaryotic genome annotation; Galaxy Version 1.14.6) and Pharokka (rapid standardized annotation tool for bacteriophage genomes and metagenomes; Galaxy Version 1.3.2). Alignment und phylogenetic tree were generated using ClustalOmega (Madeira et al., 2024).

### 2.5 Negative staining of phages

Phages are adsorbed for 15–30 s onto a carbon film and washed twice on TE buffer droplets (10 mM TRIS, 1 mM EDTA, pH 6.9) according to Dreiseikelmann et al. (2017). After washing, samples were negatively stained with 2% aqueous uranyl acetate by heat drying on a 60 W bulb after blotting excessive liquid with a filter paper. Samples were examined in a Zeiss TEM 910 transmission electron microscope (Zeiss, Oberkochen, Germany) at an acceleration voltage of 80 kV and at calibrated magnifications with a line replica. Images were recorded digitally with a slow-scan closed circuit device (CCD)-camera (ProScan, 1,024 × 1,024, Proscan Elektronische Systeme GmbH, Scheuring, Germany) with ITEM-Software (Olympus Soft Imaging Solutions GmbH, Münster, Germany). The head diameter and tail length were determined using the same software and calculating the average size from a minimum of 10 measurements.

### 2.6 Efficacy testing of phages’ bacteria reduction capability in liquid culture (planktonic killing assay)

The growth of two *S. equi* subsp. *zooepidemicus* isolates (isolates 9 and 10) with and without exposure to the respective bacteriophages (vB_SeqZP_LmqsRe26-1, vB_SeqZP_LmqsRe26-2 and vB_SeqZP_LmqsRe26-3) at different multiplicities of infection (MOI input of 1 and 10) (Danis-Wlodarczyk et al., 2021) was examined in liquid cultures using a Tecan Spark Microplate Reader (Tecan Group AG, Männedorf, Switzerland), similar to the protocol described by Steffan et al. (2021) with some modifications. Briefly, bacterial overnight cultures were adjusted to a McFarland standard of 5 in 10 mM MgSO_4_ and used to inoculate 25 mL CBHI that was then incubated for 3 h while shaking (130 rpm). Next, wells of 48-well microplates were filled with 200 μL of the culture that was adjusted to 0.5 McFarland standard and diluted in CBHI (1:100). The phage suspensions, adjusted to 1 × 10^6^ (MOI 1) and 1 × 10^7^ (MOI 10), PFU/ml in CBHI were added to the respective well. The plates were aerobically incubated with double orbital shaking at 37°C for 24 h. Optical density measurements (OD_600_) were taken hourly during incubation. Bacterial cultures inoculated with CBHI instead of phages served as positive controls. Two replicates per well were used and the experiment was performed in triplicate. The area under the curve (AUC) of OD_600_ measurements and virulence indices were calculated as described by Peh et al. (2023).

### 2.7 Efficacy testing of phages’ bacteria reduction capability in an equine endometrial explant model

Endometrial explants culture was performed according to Monteiro de Barros et al. (2021). Equine uteri and ovaries were collected at a slaughterhouse directly after slaughter of diestrous mares (*n* = 5; 3 Warmblood and 2 American Quarter Horse mares; mean age: 15.2 years (range: 5–20 years)) and transported on ice to the laboratory. The interval between exsanguination and arrival in the laboratory was between 4 to 5 h (mean: 263 min). On arrival, cycle stage was determined as described (Köhne et al., 2022). Before the next processing steps, the serosal surface of the uteri was rinsed with ethanol (70%) and uterine horns and body were opened using a sterile scalpel. Next, endometrial samples for microbial culture and cytology were collected for exclusion of endometritis. Additionally, an endometrial tissue sample comparable to a biopsy was obtained and submitted to histopathological examination. The swab was smeared directly on Columbia agar supplemented with sheep blood in three fractions and incubated (24 h, 37°C) before microbial growth was assessed. When presence of microbial growth was noted, the results of the experiment were excluded from further analysis as it was done when cytological examination using standard procedures (Dascanio, 2003) revealed presence of polymorphonuclear granulocytes.

Endometrial explants were collected using skin biopsy punches (8 mm diameter, Eickemeyer KG, Tuttlingen, Germany), sterile forceps and scissors. Directly afterwards, explants were transferred into Hanks Balanced Salt Solution (20 mL; Gibco™ HBSS, without calcium, magnesium and phenol red, Fisher Scientific GmbH, Schwerte, Germany), supplemented with amikacin sulfate (500 μg per ml medium; Briklin® Amikacin, 500 mg, Bristol-Myers Squibb, München, Germany) for 5 min. After two washing steps in unsupplemented HBSS, every explant was weighed on a sterile petri dish and the biopsies were then placed individually in wells of a 6-well plate (BioLite 6 Well Multidish, Fisher Scientific GmbH, Schwerte, Germany). An additional swab for assessment of bacterial contamination was taken and handled as described above.

The culturing of explants was performed in duplicates for every experimental group. For every uterus, a negative (culture medium without phages or bacteria) and a positive control (culture medium with bacterial suspension) were included. Briefly, culture medium (negative control: 4000 μL supplemented William’s medium (William’s Liquid E Medium, Gibco™ without phenol red, Life Technologies GmbH, Darmstadt, Germany); positive control and treatment groups: 3960 μL) was added to the wells. For samples incubated with bacteria (positive control and treatment groups), 40 μL of bacterial suspension (*S. equi* subsp. *zooepidemicus* isolate 10 adjusted to 1 × 10^7^ CFU) were added. After 1 h of incubation, specific phages were added to treatment group at two different MOIs (MOI 1: 1 × 10^7^ PFU; MOI 10: 1 × 10^8^ PFU). In controls, 40 μL of 0.9% saline solution were added and in one positive control per bacterial isolate, bacterial concentration was determined as described below.

For evaluation of explant viability, lactate dehydrogenase activity was determined in samples (2 mL) obtained after 6 and 24 h of incubation and storage at −20°C until analysis (Fuji DRI-CHEM NX500i, sysmex, Norderstedt, Germany). Moreover, histopathological examination was performed in one duplicate per group after 24 h of incubation. Endometrial biopsies were processed and analyzed as described elsewhere (Köhne et al., 2022). Examination of histological slides was performed by a trained examiner blinded to the treatment and results are reported descriptively.

For qualitative analysis of bacterial growth in negative controls, direct plating as described above and an enrichment culture in BHI liquid broth were performed. In cases of positive bacterial growth after incubation (37°C, 24 h), results of the experiment were excluded from further analysis. For quantitative analysis of bacterial growth, explants were transferred to a sterile saline (0.9%, 1,000 μL) and vortexed (3 ×; 10 s). Next, the explant was discarded and the suspension was used to determine the concentration of bacteria via a decimal dilution series. Concentrations were then calculated from the number of colony forming units (CFU) using the following formula:


$$\begin{array}{l} \frac{\mathrm{total}\;\mathrm{no}.\;\mathrm{of}\;CFU\;\left(\mathrm{all}\;\mathrm{plates}\right)}{\mathrm{no}.\;\mathrm{of}\;\mathrm{plates}\;\left(\mathrm{lowest}\;\mathrm{dilution}\right)*1+\mathrm{no}.\;\mathrm{of}\;\mathrm{plates}\;\left(2nd\;\mathrm{lowest}\;\mathrm{dilution}\right)*0.1}\\ =\mathrm{concentration}\;\left[CFU/\mathrm{ml}\right] \end{array}$$

For determining phage concentrations, 500 μL of the explant medium were filtrated, dilution series prepared and plaque assays were performed. Phage concentrations were determined in full analogy to the previously introduced method for bacterial concentrations replacing CFU by plaque forming units (PFU) in the formula above.

### 2.8 Helium ion micro-imaging of phage-bacteria interaction on the explant

At the end of one of the explant experiments, explants (treatment group and negative control) were submitted to helium ion microscopy (HIM). In preparation of the micro-imaging, the samples were fixed in glutaraldehyde (3%, dissolved in sodium-cacodylate buffer, pH 7.4) overnight at 4°C. The samples were then washed in cacodylate buffer (10 min) thrice to rinse off the fixative. Subsequently the samples were post-fixed in osmium tetroxide solution (2%) at room temperature for 2 h and thereafter rinsed thrice in distilled water (10 min). After dehydration of the samples in a graded ethanol:water series up to 75% ethanol, the samples were mailed to the Helmholtz Center for Environmental Research – UFZ, Leipzig, Germany. After completion of the ethanol series (100%), the ethanol in the samples was replaced in preparation of gentle air-drying. For that, the samples were subjected to a 1:1 mixture of ethanol and hexamethyldisilazane (HMDS, Sigma-Aldrich, Merck KGaA, Darmstadt, Germany) for 10 min and thereafter to pure HMDS for 20 min. Then the samples were placed in a dry petri dish in a fume hood to dry for 24 h. In preparation of HIM, the dried samples were mounted onto standard stubs for electron microscopy using a conductive silver epoxy glue (quick-drying conductive silver Acheson 1,415, Plano GmbH, Wetzlar, Germany). HIM imaging was done with a Zeiss Orion NanoFab (Zeiss, Peabody, MA, United States) scanning HIM using an ion-landing-energy of 25 keV, a 10-μm aperture and an Everhard-Thornley-type secondary electron detector. To achieve both high lateral resolution (≤2 nm) and a reasonable contrast, the beam current was set between 0.08 pA (high magnification) and 0.25 pA. Charge compensation during imaging was achieved with an electron flood-gun operated in line-flooding mode. In order to avoid beam damage and to allow for efficient charge compensation the dwell time of the beam on a pixel was kept between 0.5 and 1.0 μs.

### 2.9 Statistical analysis

Statistical analysis of the experimental data was performed using GraphPad Prism 9.2.0 (GraphPad Software, San Diego, United States). To meet the input requirements of the software, the bacterial concentration data (section 2.7) was calculated to log_10_ CFU/ml. For parametric data (Shapiro–Wilk test), an unpaired *t*-test was used. Data from efficacy testing of phages in liquid culture and on explants was analyzed with a Dunn’s multiple comparison test for significant differences. For analysis of effects of incubation on LDH activity, values were grouped (50 U/L, 50–100 U/L, > 100 U/L) and comparisons were made using chi-square test. For all tests, *p*-values below 0.05 were considered significant.

## 3 Results

### 3.1 Phage isolation and characterization

In total, 12 samples from horses and horse husbandries were examined for the presence of phages using the soft-agar overlay technique with clinical isolates of *S. equi* subsp. *zooepidemicus* (*n* = 6). After min. 3-fold serial purification and propagation of plaques, purified phages (*n* = 13) were obtained. Most phages were isolated from smegma of a healthy stallion (*n* = 6) and residues of the breeding barn (*n* = 4), while in other samples (drain (stallion stable and breeding barn), manure) only few phages were found (Table 1).

**Table 1:** Overview of isolated phages with name, origin, bacterial isolate number, host bacterium and isolation passage.

Phage name	Origin	Isolate no. (bacterial)	*Host bacterium*	Isolation passage
vB_SeqZ_LmqsRe241-1	Residues breeding barn	5	*S. equi* subsp. *zooepidemicus*	4
vB_SeqZ_LmqsRe238-1	Drain (stallion stable)	5	*S. equi* subsp. *zooepidemicus*	4
vB_SeqZP_LmqsRe26-1	Smegma (stallion)	9	*S. equi s*ubsp. *zooepidemicus*	4
vB_SeqZP_LmqsRe26-3	Smegma (stallion)	10	*S. equi* subsp. *zooepidemicus*	4
vB_SeqZP_LmqsRe26-2	Smegma (stallion)	10	*S. equi* subsp. *zooepidemicus*	4
vB_SeqZ_LmqsRe241-4	Residues breeding barn	13	*S. equi* subsp. *zooepidemicus*	4
vB_SeqZ_LmqsRe26-4	Smegma (stallion)	13	*S. equi* subsp. *zooepidemicus*	4
vB_SeqZ_LmqsRe235-1	Horse manure	4	*S. equi* subsp. *zooepidemicus*	8
vB_SeqZ_LmqsRe26-6	Smegma (stallion)	4	*S. equi* subsp. *zooepidemicus*	4
vB_SeqZ_LmqsRe241-2	Residues breeding barn	5	*S. equi* subsp. *zooepidemicus*	4
vB_SeqZ_LmqsRe26-5	Smegma (stallion)	11	*S. equi* subsp. *zooepidemicus*	4
vB_SeqZ_LmqsRe237-1	Drain (wash box)	11	*S. equi* subsp. *zooepidemicus*	4
vB_SeqZ_LmqsRe241-3	Residues breeding barn	13	*S. equi* subsp. *zooepidemicus*	4

**Figure 1:**
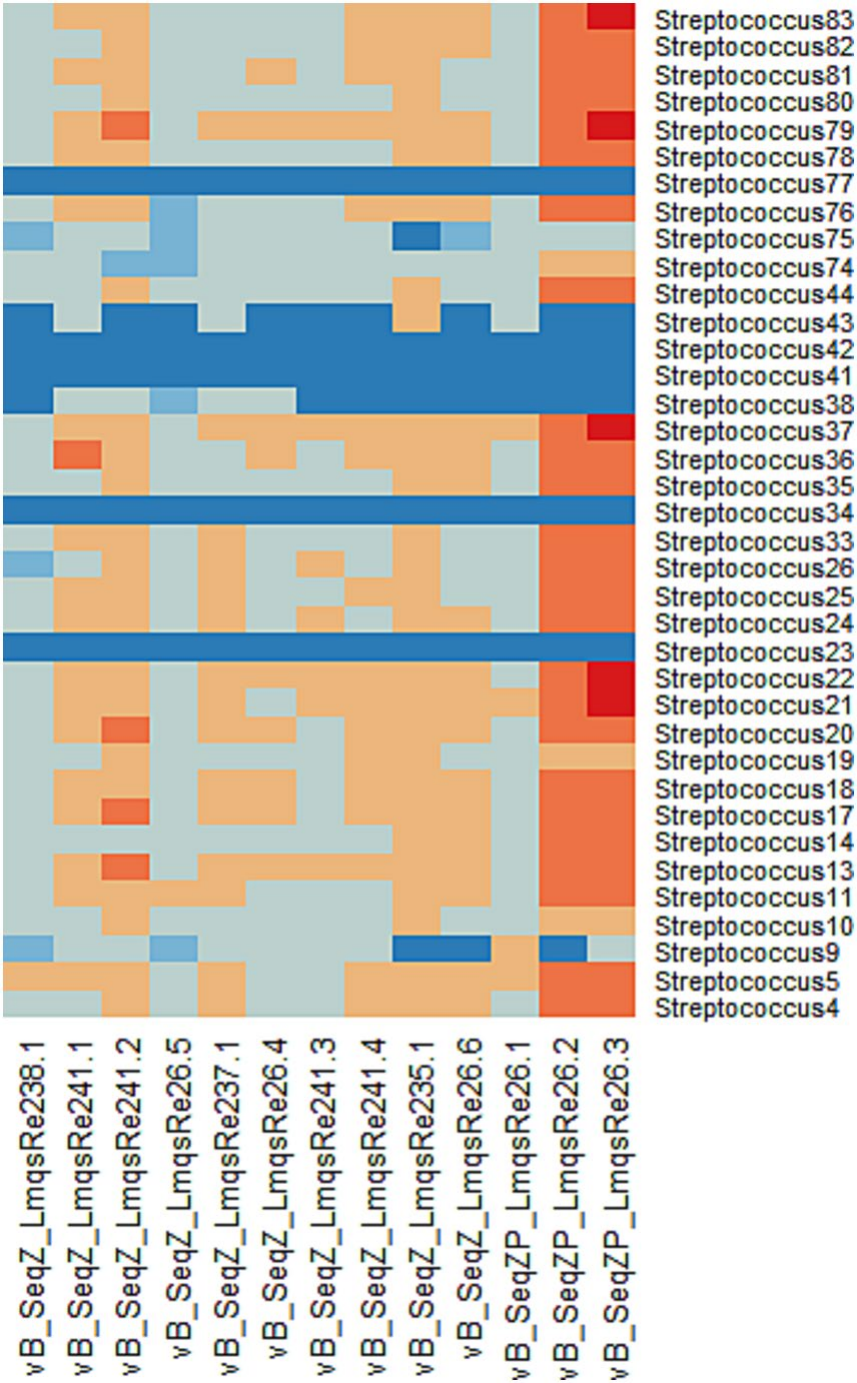
Heat map of phage host range (*Streptococcus equi* subspecies *zooepidemicus*). Phages are displayed on the x-axis and bacterial isolates on the y-axis. Dark blue: no sensitivity (no plaque formation), light blue: moderately low sensitivity (presence of an opaque plaque), grey: low sensitivity (EOP < 0.1), light orange: moderate sensitivity (0.1 ≤ EOP ≤ 1), dark orange: high sensitivity (1 < EOP ≤10), red: ultimate sensitivity (10 < EOP).

The host ranges of all purified phages were analyzed as shown in Figure 1. Phage vB_SeqZP_LmqsRe26-3 (LmqsRe26-3) showed the broadest host range, forming plaques with 30 of 37 bacterial isolates (81.1%) and phage vB_SeqZP_LmqsRe26-2 (LmqsRe26-2) with 29 of 37 isolates (78.4%). The lowest host range was shown by phages vB_SeqZ_LmqsRe235-1 and vB_SeqZ_LmqsRe26-6. All purified phages showed turbid plaque formation in at least 26 tested bacterial isolates. The two bacteriophages showing the broadest host range (LmqsRe26-3 and LmqsRe26-2) and one phage with a relatively low EOP and narrow host range (vB_SeqZP_LmqsRe26-1 (LmqsRe26-1)) were selected for further characterization.

**Figure 2:**
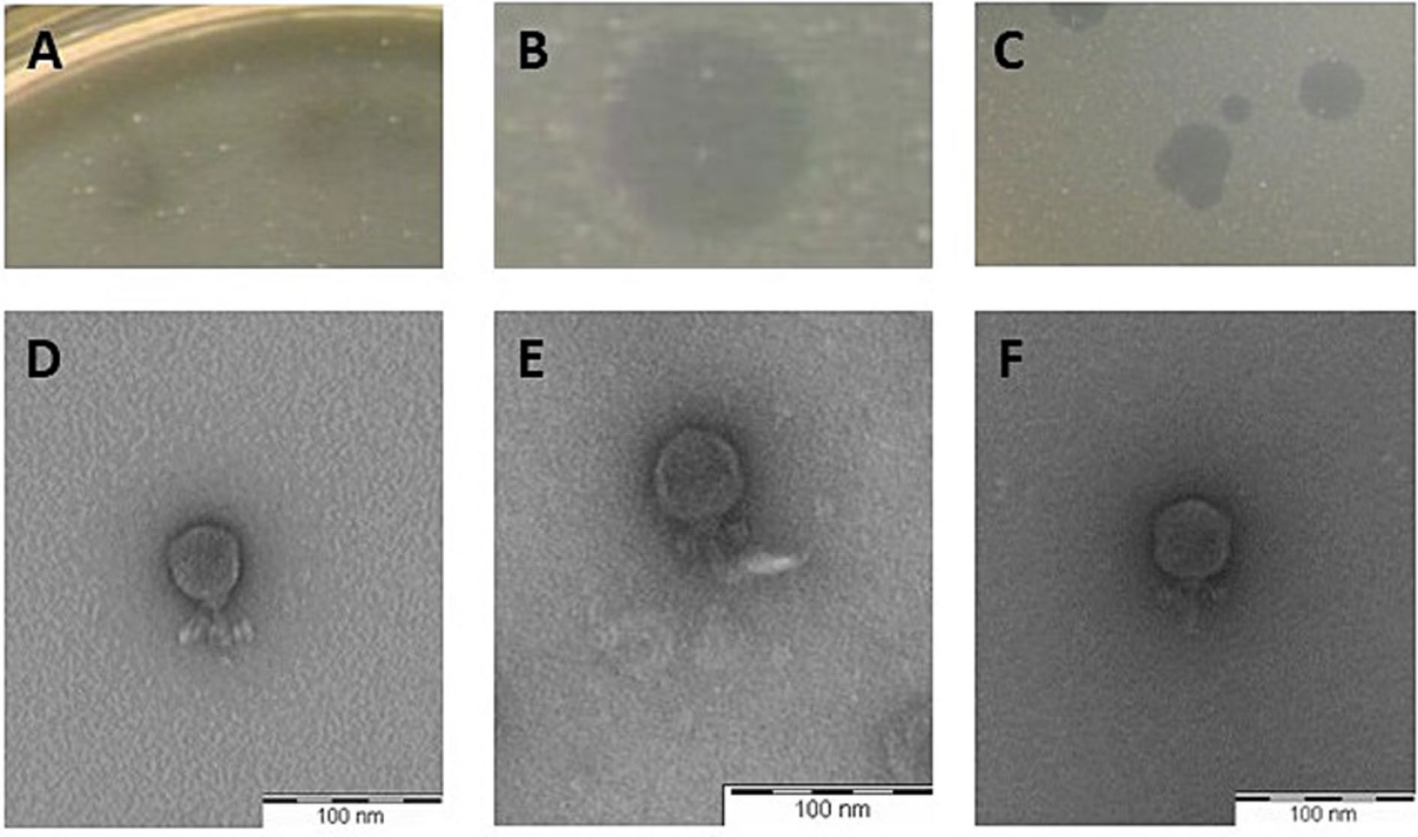
Plaque morphology of three *Streptococcus equi* subspecies *zooepidemicus*-specific phages (A–C) and transmission electron micrograph of negatively stained phages vB_SeqZP_LmqsRe26-1 (D), vB_SeqZP_LmqsRe26-2 (E) and vB_SeqZP_LmqsRe26-3 (F). Scale bar = 100 nm.

**Figure 3:**
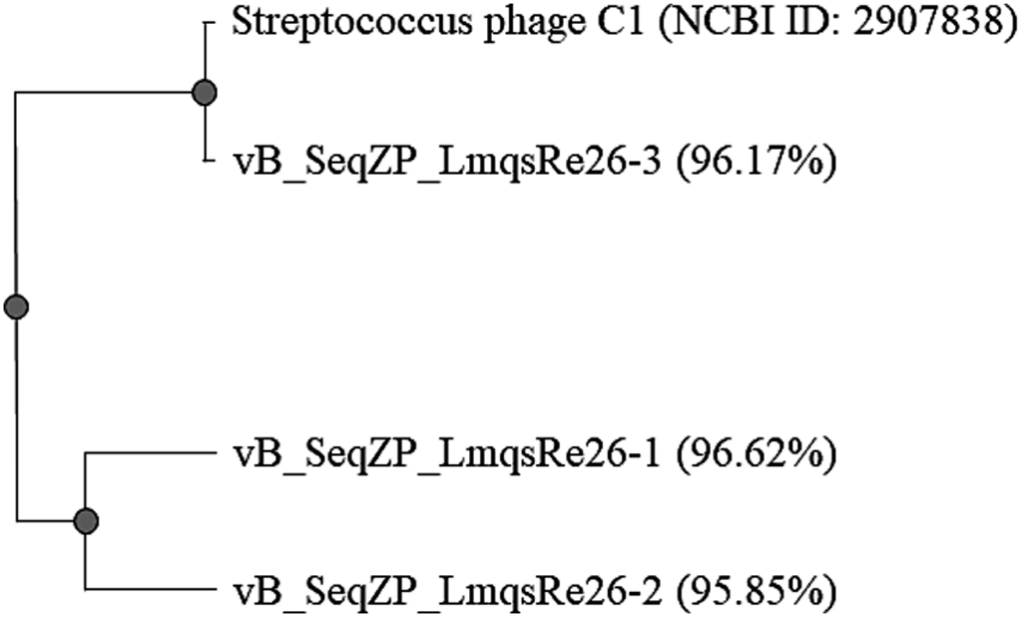
Phylogenetic analysis of newly isolated phages vB_SeqZP_LmqsRe26-1, vB_SeqZP_LmqsRe26-2, and vB_SeqZP_LmqsRe26-3 and DNA sequence identity (%) compared to the genome of *Streptococcus*-phage C1 (NCBI Nucleotide accession number: NC_004814.1) generated with Clustal Omega.

**Figure 4:**
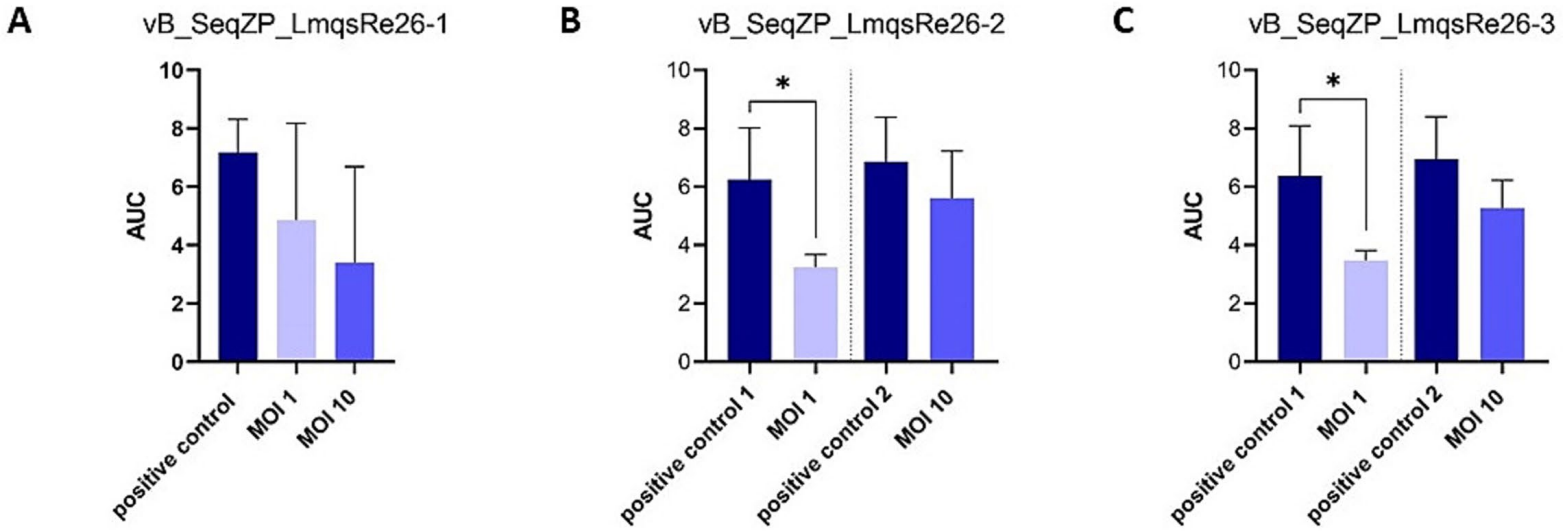
Area under the curve (optical density measurements at 600 nm; OD_600_) after incubation of *Streptococcus equi* subspecies *zooepidemicus* isolates (*n* = 2; A–C) incubated with different phages (A) vB_SeqZP_LmqsRe26-1, (B) vB_SeqZP_LmqsRe26-2, (C) vB_SeqZP_LmqsRe26-3 for 24 h. * indicates a significant difference as determined by an unpaired t-test and Dunn’s posthoc test.

**Figure 5:**
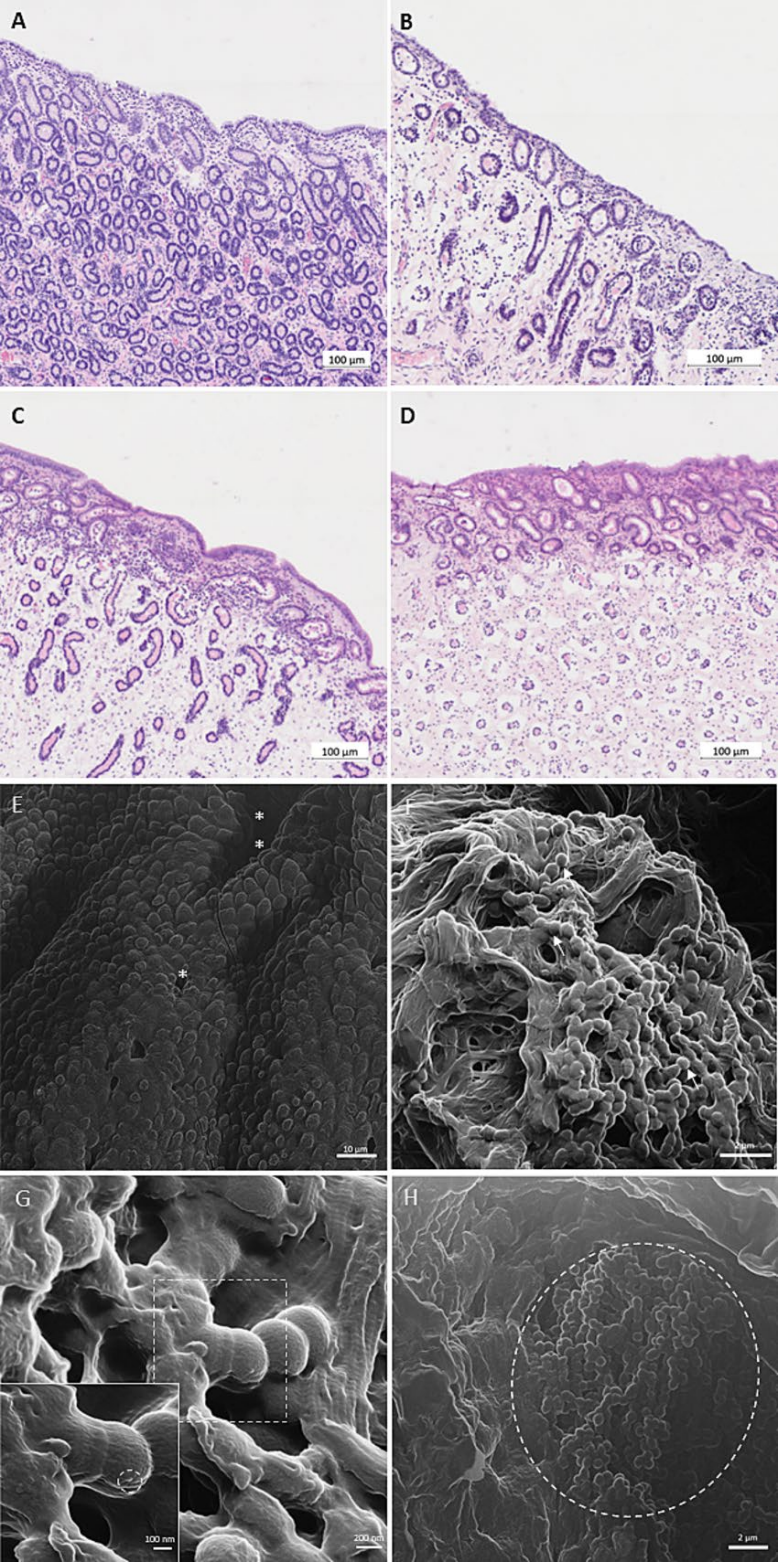
Light microscopy (A–D) and helium ion microscopy (HIM; E-H) visualization of equine endometrial explants after incubation for 24 h at 37°C. (A) HE stained endometrium (American Quarter Horse, 4 years old) after slaughter. (B) HE stained endometrium (Trakehner, 18 years old) after incubation for 24 h at 37°C in explant culture medium (negative control). (C) HE stained endometrium (Hanoverian, 18 years old) after co-incubation for 24 h at 37°C in explant culture medium with *Streptococcus (S.) equi* subspecies (subsp.) *zooepidemicus* (positive control). (D) HE stained endometrium (Trakehner, 18 years old) after co-incubation for 24 h at 37°C in explant culture medium with *S. equi* subsp. *zooepidemicus* and the specific phage vB_SeqZP_LmqsRe26-2 (multiplicity of infection (MOI): 10; treatment). Scale bars = 100 μm. (E) Negative control (HIM): incubation without bacteria or phages – note endometrial crypts (**), glandular ducts (*) and intact luminal epithelium. Scale bar = 10 μm. (F) Treatment group (HIM): incubation with *S. equi* subsp. *zooepidemicus* and the specific phage vB_SeqZP_LmqsRe26-2 at MOI 10 – note the presence of coccoid bacteria (arrows) and the complete loss of tissue architecture. Scale bar = 2 μm. (G) Treatment group (HIM): incubation with *S. equi* subsp. *zooepidemicus* and the specific phage vB_SeqZP_LmqsRe26-2 at MOI 10 – note the presence of coccoid bacteria and the adherent structure (magnification: dashed circle). Scale bar = 200 nm / 100 nm (magnification). (H) Treatment group: incubation with *S. equi* subsp. *zooepidemicus* and the specific phage vB_SeqZP_LmqsRe26-3 at MOI 10 – note the accumulation of coccoid bacteria (dashed circle) within an endometrial crypt. Scale bar = 2 μm.

The plaque morphology formed by the selected phages on their respective hosts on overlays with 0.7% agar were clear, their size ranging from 2 (LmqsRe26-2; Figure 2B) to 5 mm (LmqsRe26-3; Figure 2C) diameter, showing a slight halo (LmqsRe26-1; Figure 2A).

Negatively stained electron micrographs of these phages revealed presence of an icosahedral head (diameter: 47.4 ± 3.1 nm (LmqsRe26-1), 45.6 ± 1.4 nm (LmqsRe26-2) and 45.9 ± 2.3 nm (LmqsRe26-3)) and short tails [23.3 ± 1.6 nm (LmqsRe26-1), 25.8 ± 1.7 nm (LmqsRe26-2) and 27.3 ± 2.8 nm (LmqsRe26-3)], as observed in podoviruses (Figures 2D–F).

For further analysis, the selected phages were subjected to whole genome sequencing. All quality parameters of the prepared DNA and DNA sequencing libraries met the necessary criteria. Contigs were assembled showing genome lengths of 16,154 bp (vB_SeqZP_LmqsRe26-1), 13,280 bp (vB_SeqZP_LmqsRe26-2) and 16,165 bp (vB_SeqZP_LmqsRe26-3), representing circularly permuted chromosomes. According to blastn analysis, the phage genomes of vB_SeqZP_LmqsRe26-1 and vB_SeqZP_LmqsRe26-3 had a 96.62and 96.17% identity, respectively, with the *Streptococcus*-phage C1 (*Fischettivirus*, family *Rountreeviridae*; Nelson et al., 2003; Clark and Clark, 1927). The most likely incomplete assembly of vB_SeqZP_LmqsRe26-2 showed a 95.85% similarity to the same phage (Figure 3). Genome annotation did not reveal the presence of genes associated with virulence or resistance or lysogenic lifecycle in any of the phages. The complete genome sequences were deposited into the GenBank database with the accession numbers PQ425460 (vB_SeqZP_LmqsRe26-1) and PQ425461 (vB_SeqZP_LmqsRe26-3).

### 3.2 Bacterial reduction by phages in liquid culture

A planktonic killing assay was conducted for determining the ability of phages to inhibit bacterial growth of the strains *S. equi* subsp. *zooepidemicus* 9 (LmqsRe26-1) and 10 (LmqsRe26-2 and LmqsRe26-3) in liquid culture. For this purpose, the host bacteria strains – alone or in combination with phages at different multiplicities of infection (MOIs) – were incubated for 24 h. In order to perform online cell-number measurements, the optical density (OD_600_) was measured in parallel using a Tecan Spark Multiplate reader. Significant bacterial reduction compared to phage-free growth controls was observed at MOI 1 of LmqsRe26-2 and LmqsRe26-3, but not for LmqsRe26-1 (Figures 4A–C). At MOI 10 any significant effect was found for none of the selected phages. The virulence indices, calculated from the AUC of phage treatments and growth controls, were highest for LmqsRe26-1 at MOI 10 (52.2) and lowest for LmqsRe26-2 at MOI 10 (12.8), but, interestingly, were higher for LmqsRe26-2 and LmqsRe26-3 at MOI 1 (49.7 and 46.9) as compared to MOI 10 (12.8 and 19.3; Table 2).

**Table 2:** Virulence indices as determined by a planktonic killing assay for phages specific to *Streptococcus equi* subspecies *zooepidemicus* in different multiplicities of infection (MOIs).

Phage	Multiplicity of infection	Virulence index
vB_SeqZP_LmqsRe26-1	1	32.3
	10	52.2
vB_SeqZP_LmqsRe26-2	1	49.7
	10	12.8
vB_SeqZP_LmqsRe26-3	1	46.9
	10	19.3

### 3.3 Viability of explants

Explant cultures of endometrial tissue samples were incubated to provide an *ex vivo* model for investigation of phage-bacteria interactions. In total, six explant experiments were conducted for 24 h each. Preliminary results of one trial were excluded from further analysis due to bacterial contamination of the uterus. Functionality and vitality of the explant was assessed by histopathological examination of the explant and LDH activity. Histopathological analysis was performed before the experiment and after incubation. Analysis of specimens taken at arrival in the laboratory (before incubation) revealed occasional presence of periglandular fibrosis, disseminated glandular nests and glandular atrophy, indicating mild to moderate degeneration of the slaughtered mares’ endometrium (Figure 5A). No signs of endometritis were detected. After incubation for 24 h, parts (up to 90%) of the luminal epithelium were either completely lost or epithelial cells appeared cuboidal and had lost their cilia (Figure 5B). Changes were more pronounced for explants co-incubated with bacteria, where the epithelial cells were absent in the majority of explants (Figure 5C), while in the control explants without co-incubation of bacteria, signs of epithelial degeneration were less pronounced. Epithelial destruction was unaffected by presence of phages (Figure 5D). Loss of typical endometrial tissue architecture – including the uterine glands – was observed in some explants, while others showed a nearly intact tissue structure, regardless of being incubated with or without bacteria (Figures 5B–D). No significant impact of time or treatment on LDH activity was detected after 6 and 24 h of incubation (*p* > 0.05; Supplementary Table S2).

### 3.4 Bacterial reduction efficacy of phages in an explant model

**Figure 6:**
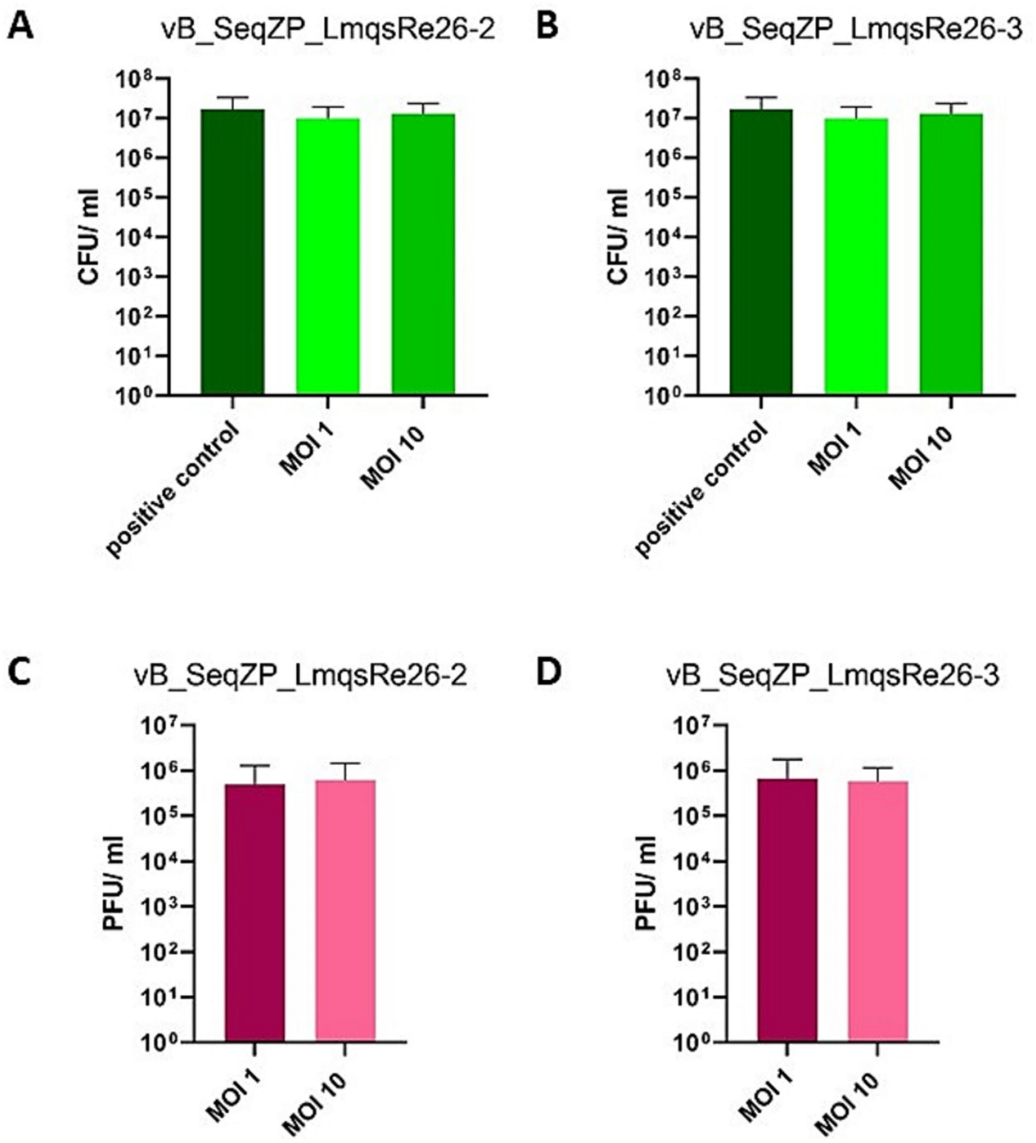
Concentration of colony forming units (CFU/ ml) of *Streptococcus equi* subspecies *zooepidemicus* (A,B) after incubation (24 h) on an equine endometrial explant in culture after infection with the specific phages vB_SeqZP_LmqsRe26-2 (A) and vB_SeqZP_LmqsRe26-3 (B) at two different multiplicities of infection (MOI 1 and MOI 10). Positive controls of bacteria were cultured without phages. Concentration (plaque forming units (PFU)/ml) of specific phages [vB_SeqZP_LmqsRe26-2 (C) and vB_SeqZP_LmqsRe26-3 (D)] after co-incubation (24 h) with *Streptococcus equi* subspecies *zooepidemicus* on an equine endometrial explant at two different multiplicities of infection (MOI 1 and MOI 10).

The efficacy of two phages (LmqsRe26-2 and LmqsRe26-3) for reduction of their host isolate *S. equi* subsp. *zooepidemicus* 10 was evaluated in the explant model as single phage assays. After 24 h of co-incubation, no significant effect of LmqsRe26-2 was observed with any of the tested MOIs (control: 1.71 × 10^7^ ± 1.67 × 10^7^ CFU/mL vs. MOI 1: 9.89 × 10^6^ ± 9.21 × 10^6^ CFU/ ml vs. MOI 10: 1.30 × 10^7^ ± 1.1 × 10^7^ CFU/mL; Figure 6A). Using LmqsRe26-3, bacterial growth was not significantly reduced either, however, a slight difference was measured in comparison to the untreated control (control: 1.71 × 10^7^ ± 1.67 × 10^7^ CFU/mL vs. MOI 1: 1.37 × 10^7^ ± 1.01 × 10^7^ CFU/mL vs. MOI 10: 1.51 × 10^7^ ± 1.15 × 10^7^ CFU/mL; Figure 6B). The phage concentration in the medium after 24 h of incubation was 4.93 × 10^5^ ± 7.76 × 10^5^ PFU/ml (MOI 1) and 6.17 × 10^5^ ± 8.28 × 10^5^ PFU/ml (MOI 10) for LmqsRe26-2 (Figure 6C) and 6.61 × 10^5^ ± 1.09 × 10^6^ PFU/ml (MOI 1) and 5.83 × 10^5^ ± 5.66 × 10^5^ PFU/ml (MOI 10) for LmqsRe26-3 (Figure 6D), respectively.

### 3.5 Helium ion microscopy image analysis

After 24 h of incubation, explants without bacterial inoculate showed polygonal luminal epithelial cells with microvilli, glandular ducts and endometrial crypts (Figure 5E) in the HIM. The same technique revealed that co-incubation with phages and bacteria resulted in a complete loss of tissue architecture (Figure 5F) and presence of coccoid bacteria on the explant surface, especially within the endometrial crypts (Figure 5H). Occasional adherence of small particles to bacterial cells may suggest that phages adhered to the bacterial surface (Figure 5G; Supplementary Figures S1–S4).

## 4 Discussion

The isolation and characterization of phages specific to the “emerging pathogen” (Kuchipudi et al., 2021) and potentially pathogenic *S. equi* subsp. *zooepidemicus* (Kim et al., 2022) closes a gap in veterinary phage research as characterized phages specific for this important veterinary and zoonotic pathogen have not been described previously to the best of our knowledge. However, Tiwari and Timoney (2009) tested *S. equi* subsp. *zooepidemicus* isolates with the *S. equi* subsp*. equi* phage SeP9, finding no sensitivity to this phage. In 2018, Harhala et al. (2018) reported that only 3% of all published phage genomes in the NCBI (National Center of Biotechnology Information) nucleotide database were associated with phages specific to streptococci species, which has decreased to 1.3% according to NCBI blast at time of publishing. However, streptococcal infections are causing severe diseases in domestic animals (Fulde and Valentin-Weigand, 2013) and particularly *S. equi* subsp. *zooepidemicus* is a major cause for equine infertility as it has been associated with endometritis, early pregnancy loss, and placentitis in mares (LeBlanc and Causey, 2009; Rose et al., 2018; Macpherson, 2005) as well as infections in humans (Kittang et al., 2017; Eyre et al., 2010; Friederichs et al., 2010; Pelkonen et al., 2013). Thus, and due to the need to develop new non-antibiotic treatment approaches, the present study was performed.

Isolation and purification of phages from horse- and horse husbandry-associated samples resulted in the detection of 16 phages specific to *S. equi* subsp. *zooepidemicus* isolates originating from horses. All isolated phages showed EOPs of 0.1 to 1 on at least one clinical bacterial isolate. For the phages LmqsRe26-2, LmqsRe26-3, vB_SeqZ_LmqsRe241.1, and vB_SeqZ_LmqsRe241.2 even higher EOPs (between 1 and 10) were observed when plated on some isolates. Moreover, LmqsRe26-2 and LmqsRe26-3 showed the broadest host ranges, lysing around 80% of the examined *Streptococcus* isolates. As no *S. equi* subsp. *zooepidemicus* phage characterization has been published up to now, their host ranges can only be compared with other streptococci phages. In cattle-associated *S. agalactiae* phages, the broadest lytic activity observed was 65.1% (Bai et al., 2013). In isolates of the *Streptococcus bovis*/*equi* complex (SBSEC), host ranges stretched from 15.4 to 35.3% in isolates originating from bovine species and 64.3 to 71.4% in caprine isolates (Park et al., 2023). Therefore, the host range presented in this study can be considered broad and therefore promising for further therapeutic use. Although the bacterial isolates used in this study did not underwent sequence typing, but the bacterial isolates differed according to their origin and growth characteristics and phage susceptibility. Therefore, the host range determination results have to be interpreted cautiously, but genetic variance in *S. equi* subsp. *zooepidemicus*-isolates from clinical samples has been reported to be large in other studies (Preziuso et al., 2019; Retamar et al., 2024).

Electron microscopy of the three selected phages revealed the presence of an icosahedral head size of 46.9–49.7 nm in diameter and short tails (23.3–27.3 nm), characteristic for podoviruses (Ackermann and Prangishvili, 2012). Genomic analysis revealed that the newly isolated phage (LmqsRe26-1) belongs to the genus *Fischettivirus*, family *Rountreeviridae* (Walker et al., 2022). The genome of LmqsRe26-1 was highly identical (96.62% identity) with the first described streptococcal phage (C_1_), which was isolated in 1927 from a sewage plant sample (Clark and Clark, 1927) and demonstrated lytic activity against any group C streptococci (Lancefield, 1932). It has also been noted that the C_1_ phage had lytic activity against streptococci that were not sensitive to the phage itself – a phenomenon described as “nascent lysis” in 1934 (Evans, 1934) and which has been attributed to the presence of phage-encoded endolysins later (Abdelrahman et al., 2021). While susceptibility and plaque formation was shown for the bacterial strain in planktonic killing assays, low EOP in other isolates and narrow host range could explain our results. Small host range including low EOP results might imply that even small adaptions by the bacterial strain could circumvent phage infection, e.g., in planktonic killing assays. However, further studies would be necessary to verify this hypothesis. Interestingly, the phage LmqsRe26-3, showing lower (96.17%) identity with C_1_, was even more efficient in performing lysis on a broad range of host isolates as compared to the phage LmqsRe26-1. Thus, a better adaptation to *S. equi* subsp. *zooepidemicus* of LmqsRe26-3 compared to LmqsRe26-1 can be assumed. Despite the high similarity and repeated sequencing and analysis of LmqsRe26-2 no complete genome could be assembled, in contrast to the other phages. There are several possible reasons for this finding including high microdiversity of phage LmqsRe26-1, or highly repetitive regions or regions in the genome and regions with extremely high or low coverage that prevent alignment of related phage contigs (Shen and Millard, 2021; Boeckman et al., 2024).

In the planktonic killing assay, LmqsRe26-2 and LmqsRe26-3 significantly reduced bacterial growth, while LmqsRe26-1 did not. The significant growth reduction was only observed after incubation for 24 h at MOI 1 but not at MOI 10. Loc Carrillo et al. (2005) have also reported that phages are more efficient at lower MOIs for *Campylobacter jejuni* phages. Nang et al. (2023) hypothesized that a higher phage concentration leads to higher selection pressure on phage resistance and therefore induces beneficial mutations in some bacterial cells. Possible resistance mechanisms include the loss of phage receptors on the cell surface or physical barriers, covering the phage receptor molecules (Hyman and Abedon, 2010). As bacterial isolates were neither sequenced before and after phage contact nor testing for resistances was performed after growth reduction experiments, we cannot answer this question. However, a variety of bacterial anti-phage resistance mechanisms have been reported in other streptococci species, including mutations in the methionine aminopeptidase gene (Labrie et al., 2019), activation of CRISPR-Cas systems, and the production of membrane vesicles enabling the scavenge of phage particles from the environment (Beerens et al., 2021).

To examine the *in vitro* efficient LmqsRe26-2 and LmqsRe26-3 in an environment resembling the clinical conditions in the mare, they were tested in an explant model of the equine endometrium.

Although the explants remained functional for the duration of the experiment as shown by histopathology and LDH analysis, co-incubation with *S. equi* subsp. *zooepidemicus* 10 and LmqsRe26-2 and LmqsRe26-3 resulted in alterations of the endometrium, including loss of tissue architecture and the epithelium. Since histopathological analysis of explants incubated with solely bacteria or a combination of bacteria and phages did not differ with regards to the degree of epithelial loss, we did not include explants incubated either with bacteria or phages into HIM analysis. The destructive effect of the bacteria on the endometrium is likely due to exotoxins, e.g., hemolysin, which are produced and secreted by *S. equi* subsp. *zooepidemicus* (Wittenbrink et al., 1997). This effect could have been potentiated by the fact that *S. equi* subsp. *zooepidemicus* better binds to damaged endometrial cells (Ferreira-Dias et al., 1994a).

Some of our results as well as findings in previous studies point toward a lack of phage-bacteria interaction as a reason for a lacking phage efficacy in this model: First, the altered endometrium could have contributed to a lacking efficacy of LmqsRe26-2 and LmqsRe26-3 if the binding of bacteria to the endometrium impairs the phage-bacteria interaction. However, further experiments are needed to prove this hypothesis. Second, Balcão et al. (2022) observed that the application of a phage cocktail against *E. coli* inhibited bacterial growth in an explant model of the canine endometrium for 8 h, however, after 8 h, bacterial growth normalized. The authors discussed that bacterial host cells could have been embedded in the endometrial crypts and glands, thus impairing the binding of phages to bacterial cells. In the present study, this hypothesis is supported by the fact that bacterial colonies were clustered in uterine glands as visualized by helium ion microscopy (Figure 5H). A third explanation for missing phage-bacteria interaction in this model may be related to chosen MOIs in the experiment, which might have been too low for enabling phage-bacteria interaction in the relatively large volume of the well since the phage concentration was 4 × 10^5^ (MOI 1) or 4 × 10^6^ (MOI 10) per well, respectively. Moreover, it was not possible to standardize the explant size in a way that only the mucosal surface was covered with a thin fluid film, where phages and bacteria could have been deposited to minimize diffusion within the culture medium. Interestingly, the phage concentration was decreased by two log levels as compared to the bacterial concentration at the end of the experiment, which supports the hypothesis that no phage replication occurred or phage degradation exceeded replication. Despite the missing efficacy of phages in this experiment, a volume adapted phage concentration within the model shows potential for future experiments.

HIM was performed to visualize the explant surface and possible interactions of phages and bacteria. Images of the negative control showed polygonal epithelial cells with microvilli as described for unaltered endometrium visualized by electron microscopy (Ferreira-Dias et al., 1994b). However, loss of the regular epithelial architecture was observed after co-incubation with phages and bacteria as mentioned above. In this sample, phage-like particles were detected on bacterial cells (e.g., as displayed in Figure 5G) whose structure fits the head size of phage LmqsRe26-2 as calculated by TEM analysis. This particle was closely attached to the bacterial cell membrane as described for a podovirus infection in a previous study (Casjens and Molineux, 2012). The shape of the structure is not clearly icosahedral, however, is likely due to the long HIM sample preparation routine, compared to negative stain TEM imaging. For example, HIM images of T4 phages, which also possess icosahedral heads, have shown variable shapes during infections of bacterial cells (Leppänen et al., 2017). Moreover, the presence of extracellular matrix around the bacteria could mask the phage structure. With the same probability, the visualized structure may be a bacterial vesicle during the process of endo- or exocytosis. However, extracellular vesicles have not yet been described for *S. equi* subsp. *zooepidemicus*, while being observed in *S. pneumoniae* (Mehanny et al., 2020). Further experiments are needed to clearly visualize the phage infection of bacteria in the *ex vivo* model.

In conclusion, this paper describes the isolation and characterization of phages against *S. equi* subsp. *zooepidemicus* for the first time. A relatively broad host range and high efficacy against potential host bacteria *in vitro* emphasizes their therapeutic potential. However, while reduction has been observed in planktonic killing assays, no phage-induced bacterial growth reduction was observed in an endometrial explant model, demonstrating the need for further studies on potential phage administration (e.g., optimization of MOIs and combined administration with antibiotics) and kinetics since the available results do not currently support the therapeutic use of the phages.

## Data Availability

The original contributions presented in the study are included in the article/Supplementary material. The complete genomes of the isolated phages can be found under the accession number PQ425460 and PQ425461 in the NCBI database (https://www.ncbi.nlm.nih.gov/).

## Supplementary material

Supplementary Table S1 Information on bacterial isolates (isolate number, bacterial species, origin of bacterial sample).

Supplementary Table S2 Number of explants per group (negative control, positive control, treatment group) with results of lactate dehydrogenase (LDH) activitity-analysis in explant culture medium (< 50 U/l; 50-100 U/l; > 100 U/l) after 6 and 24 h of incubation. No significant differences among treatment groups were determined by chi-square testing.

Supplementary Figures S1–S4 Additional helium ion microscopic images of an equine endometrial explant after incubation with *S. equi* subsp. *zooepidemicus* and the specific phage vB_SeqZP_LmqsRe26-2 at MOI 10 with particles suspected of being phages.
