# Supplementary figures and images for: Isolation and characterization of bacteriophages specific to Streptococcus equi subspecies zooepidemicus and evaluation of efficacy ex vivo
Source: Front Microbiol. 2024 Oct 28;15:1448958. doi: 10.3389/fmicb.2024.1448958 (PMC11550937; doi:10.3389/fmicb.2024.1448958)

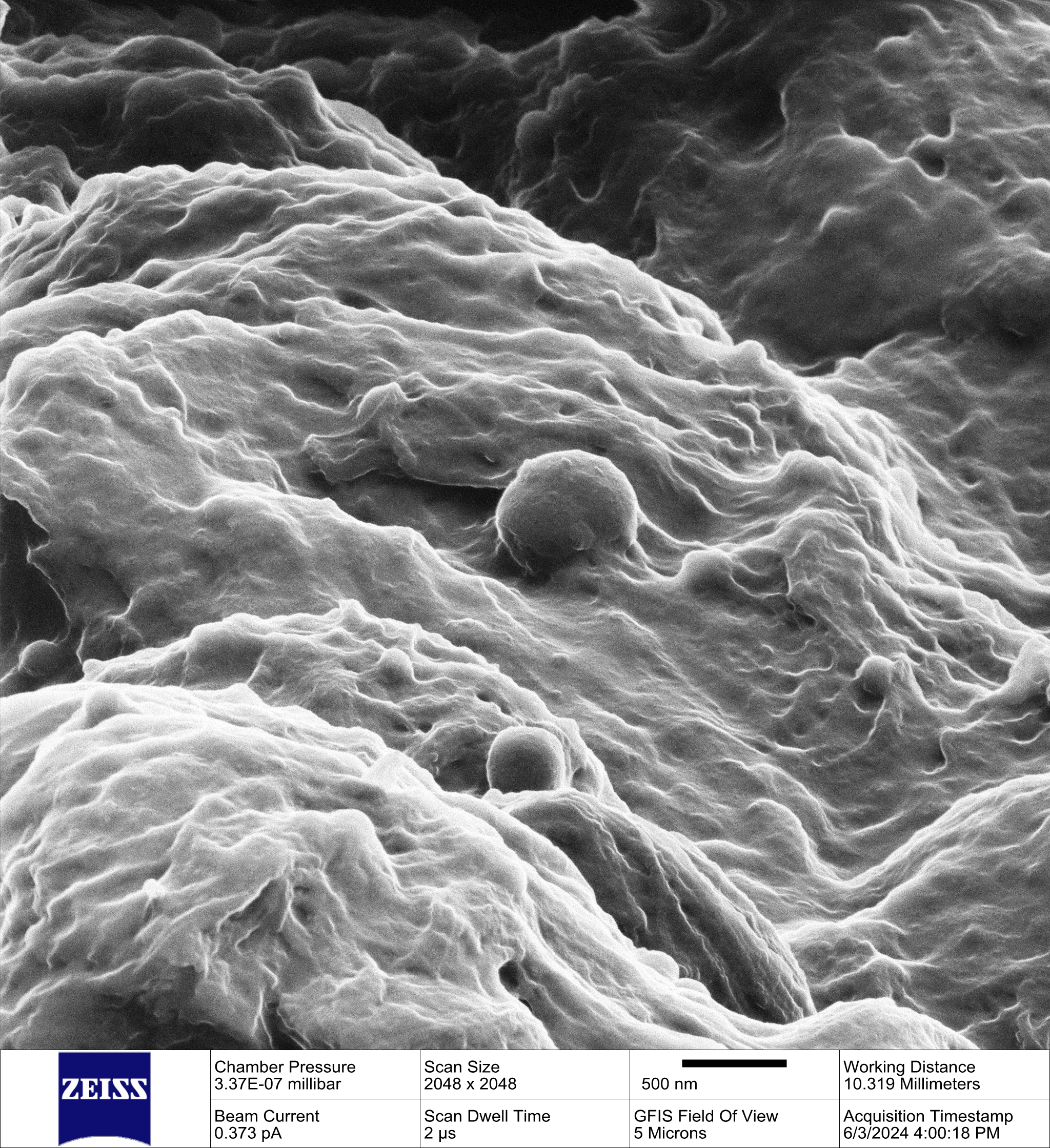

Supplement: Supplementary Table S1 — Information on bacterial isolates (isolate number, bacterial species, origin of bacterial sample). [file Image_1.TIF]

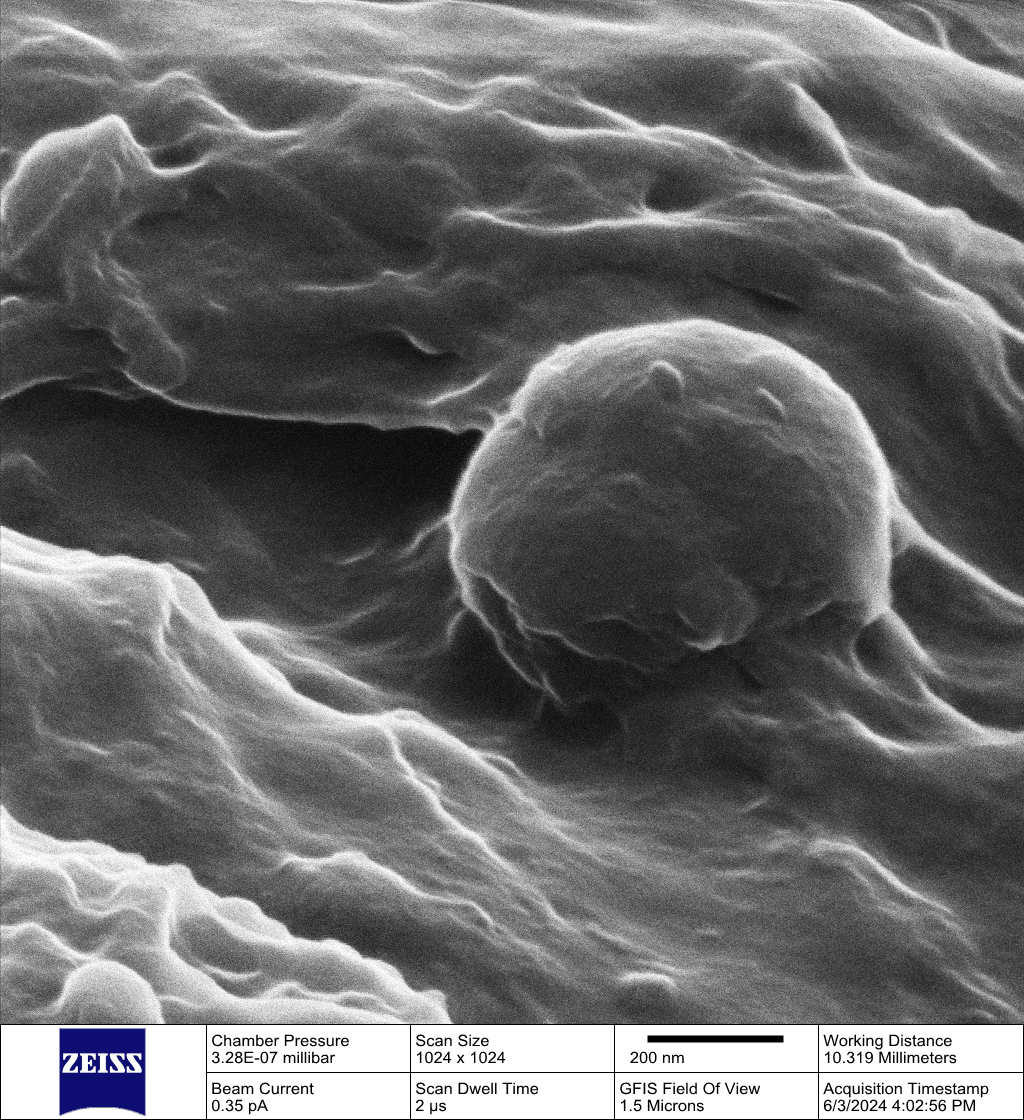

Supplement: Supplementary Table S2 — Number of explants per group (negative control, positive control, treatment group) with results of lactate dehydrogenase (LDH) activitity-analysis in explant culture medium (< 50 U/l; 50-100 U/l; > 100 U/l) after 6 and 24 h of incubation. No significant differences among treatment groups were determined by chi-square testing. [file Image_2.TIF]

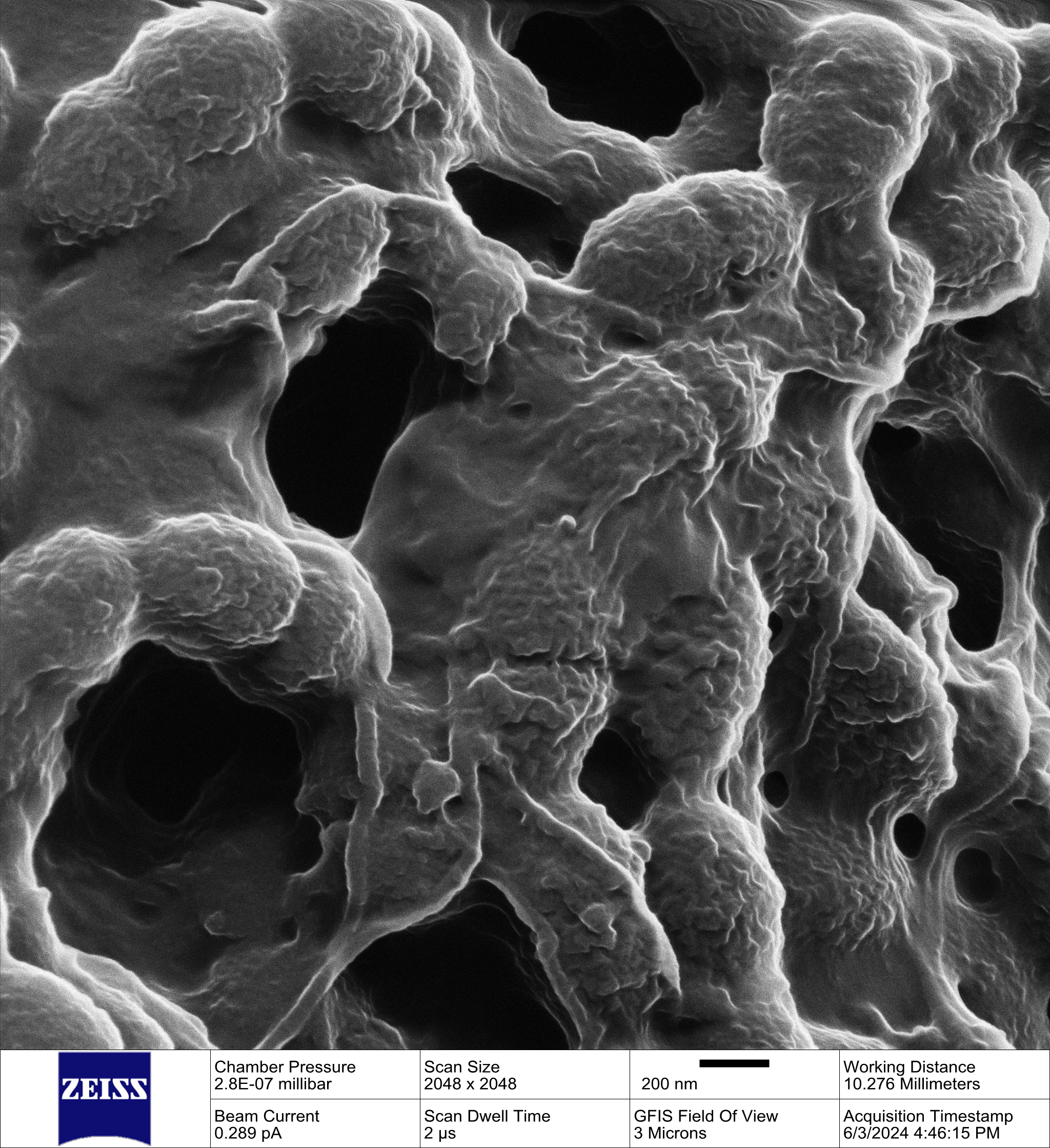

Supplement: Supplementary Figures S1–S4 — Additional helium ion microscopic images of an equine endometrial explant after incubation with S. equi subsp. zooepidemicus and the specific phage vB_SeqZP_LmqsRe26-2 at MOI 10 with particles suspected of being phages. [file Image_3.TIF]

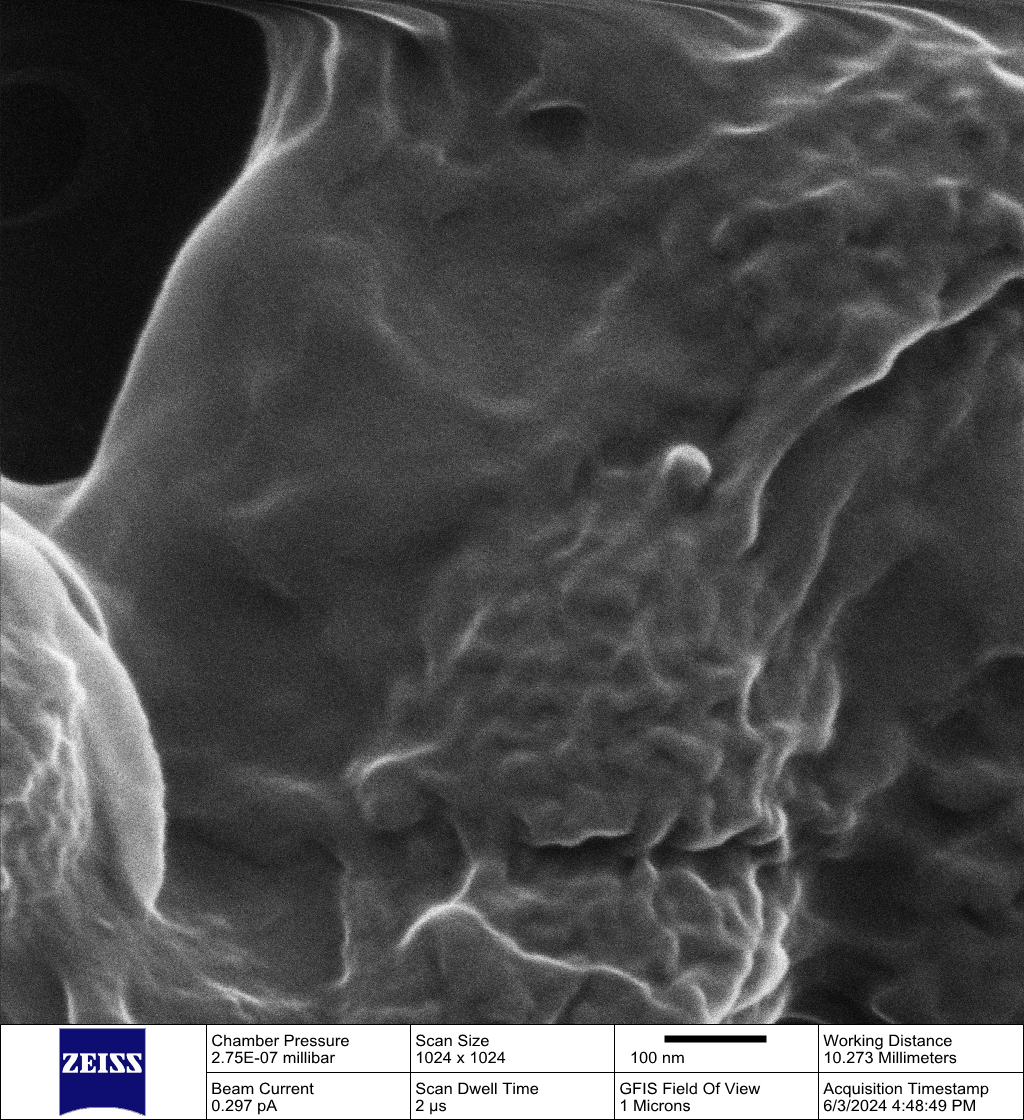

Supplement: Supplementary file 4 [file Image_4.TIF]
