# Supplementary material for: Isolation and characterization of bacteriophages specific to Streptococcus equi subspecies zooepidemicus and evaluation of efficacy ex vivo
Source: Front Microbiol. 2024 Oct 28;15:1448958. doi: 10.3389/fmicb.2024.1448958 (PMC11550937; doi:10.3389/fmicb.2024.1448958)
Supplement: Supplementary file 5 [file Table_1.DOCX]

| Bacterial  Isolate (number) | Bacterial species | Origin of bacterial sample |
| --- | --- | --- |
| 4 | *S. equi* subsp*. zooepidemicus* | cervix, mare |
| 5 | *S. equi* subsp. *zooepidemicus* | cervix, mare |
| 9 | *S. equi* subsp*. zooepidemicus* | tracheobronchial secretion, horse |
| 10 | *S. equi* subsp. *zooepidemicus* | unknown |
| 11 | *S. equi* subsp*. zooepidemicus* | sinusal secretion, horse |
| 13 | *S. equi* subsp. *zooepidemicus* | endometrium, mare |
| 14 | *S. equi* subsp*. zooepidemicus* | tracheobronchial secretion, horse |
| 17 | *S. equi* subsp. *zooepidemicus* | cervix, mare |
| 18 | *S. equi* subsp*. zooepidemicus* | cervix, mare |
| 19 | *S. equi* subsp. *zooepidemicus* | cervix, mare |
| 20 | *S. equi* subsp*. zooepidemicus* | cervix, mare |
| 21 | *S. equi* subsp. *zooepidemicus* | cervix, mare |
| 22 | *S. equi* subsp*. zooepidemicus* | cervix, mare |
| 23 | *S. equi* subsp. *zooepidemicus* | cervix, mare |
| 24 | *S. equi* subsp*. zooepidemicus* | cervix, mare |
| 25 | *S. equi* subsp. *zooepidemicus* | cervix, mare |
| 26 | *S. equi* subsp*. zooepidemicus* | mare |
| 33 | *S. equi* subsp. *zooepidemicus* | wound secretion, horse |
| 34 | *S. equi* subsp*. zooepidemicus* | umbilical abscessation, horse |
| 35 | *S. equi* subsp. *zooepidemicus* | synovia, horse |
| 36 | *S. equi* subsp*. zooepidemicus* | wound secretion, horse |
| 37 | *S. equi* subsp. *zooepidemicus* | guttural pouch, horse |
| 38 | *S. equi* subsp*. zooepidemicus* | wound secretion, horse |
| 41 | *S. equi* subsp. *zooepidemicus* | tracheobronchial secretion (horse) |
| 42 | *S. equi* subsp*. zooepidemicus* | umbilical abscessation, horse |
| 43 | *S. equi* subsp. *zooepidemicus* | wound secretion, horse |
| 44 | *S. equi* subsp*. zooepidemicus* | tracheobronchial secretion, horse |
| 74 | *S. equi* subsp. *zooepidemicus* | cervix, mare |
| 75 | *S. equi* subsp*. zooepidemicus* | cervix, mare |
| 76 | *S. equi* subsp. *zooepidemicus* | cervix, mare |
| 77 | *S. equi* subsp*. zooepidemicus* | cervix, mare |
| 78 | *S. equi* subsp. *zooepidemicus* | cervix, mare |
| 79 | *S. equi* subsp*. zooepidemicus* | cervix, mare |
| 80 | *S. equi* subsp. *zooepidemicus* | cervix, mare |
| 81 | *S. equi* subsp*. zooepidemicus* | genital swab, mare |
| 82 | *S. equi* subsp. *zooepidemicus* | genital swab, mare |
| 83 | *S. equi* subsp. *zooepidemicus* | genital swab, mare |
