# Supplementary material for: Isolation and characterization of bacteriophages specific to Streptococcus equi subspecies zooepidemicus and evaluation of efficacy ex vivo
Source: Front Microbiol. 2024 Oct 28;15:1448958. doi: 10.3389/fmicb.2024.1448958 (PMC11550937; doi:10.3389/fmicb.2024.1448958)
Supplement: Supplementary file 6 [file Table_2.DOCX]

| LDH | Negative control | | Positive control | | Treatment group | |
| --- | --- | --- | --- | --- | --- | --- |
|  | 6 h | 24 h | 6 h | 24 h | 6 h | 24 h |
| < 50 U/l | 11  (78.6%) | 7  (46.7%) | 21 (91.3%) | 13 (54.2%) | 54  (80.6%) | 30 (45.5%) |
| 50 – 100 U/l | 3  (21.4%) | 7  (46.7%) | 2  (8.7%) | 7  (29.2%) | 13  (19.4%) | 23 (34.9%) |
| > 100 U/l | 0  (0%) | 1  (6.7%) | 0  (0%) | 4  (16.7%) | 0  (0%) | 13 (19.7%) |
| number | 14 | 15 | 23 | 24 | 67 | 66 |
